# On call at the mall: a mixed methods study of U.S. medical malls

**DOI:** 10.1186/1472-6963-13-471

**Published:** 2013-11-09

**Authors:** Lori Uscher-Pines, Ateev Mehrotra, Ramya Chari

**Affiliations:** 1 RAND Corporation, 1200 S Hayes St, Arlington, VA 22202, USA; 2 Boston MA and Harvard Medical School, RAND Corporation, Boston, MA, USA

**Keywords:** Healthcare delivery, Medical mall, Access to care, Disparities

## Abstract

**Background:**

The decline of the traditional U.S. shopping mall and a focus on more consumer- centered care have created an opportunity for “medical malls”. Medical malls are defined as former retail spaces repurposed for healthcare tenants or mixed-use medical/retail facilities.

We aimed to describe the current reach of healthcare services in U.S. malls, characterize the medical mall model and emerging trends, and assess the potential of these facilities to serve low-income populations.

**Methods:**

We used a mixed methods approach which included a comprehensive literature review, key informant interviews, and a descriptive analysis of the Directory of Major Malls, an online retail database.

**Results:**

Six percent (n = 89) of large, enclosed shopping malls in the U.S. include at least one non-optometry or dental healthcare tenant. We identified a total of 28 medical malls across the U.S., the majority of which opened in the past five years and serve middle or high income populations. Stakeholders felt the key strengths of medical malls were more convenient access including public transportation, greater familiarity for patients, and “one stop shopping” for primary care and specialty services as well as retail needs.

**Conclusions:**

While medical malls currently account for a small fraction of malls in the US, they are a new model for healthcare with significant potential for growth.

## Background

Shopping malls are not traditional settings for healthcare delivery, but that may be changing. As economic trends threaten the long-term viability of large, enclosed shopping centers, mall developers are increasingly looking to new and innovative uses for existing spaces [1]. Over the last 20 years, shopping malls across the United States have experienced increasing rates of vacancy, and 19% of the 2,000 regional malls (defined in the mall industry as containing 300,000+ square feet of shopping space and at least one major department store) [2] in the United States are dead or dying [1,3-5]. The decline of shopping malls is occurring at the same time that healthcare needs are growing and medical care is becoming more consumer-centric. For example, the focus on greater convenience for patients is illustrated by the interest in retail clinics, same-day scheduling in physicians’ offices, and after-hours care. As such, many hospital and physician groups view underutilized mall spaces as attractive options for locating healthcare services and bringing services closer to their customers. In fact, the last 20 years has witnessed the growth of the “medical mall” model, where healthcare organizations repurpose an entire mall or lease space within a mall to create a mixed-use medical/retail facility [6].

The medical mall model offers many potential benefits to both patients and providers. It is consumer-centric due to the convenient locations, ample parking, extended hours, easy access to non-medical services, and a one-stop healthcare experience for specialty and primary care services. In addition, hospitals or physicians in medical malls may benefit from expanded clinical and office space, higher visibility in the communities served, and an increased patient referral base [7]. In low-income areas, medical malls may be designed to both increase access to healthcare and act as an engine of neighborhood and economic revitalization. Jackson Medical Mall in Jackson, Mississippi, is one such example of a medical mall serving a disadvantaged, primarily African-American community with the dual goal of improving healthcare and stimulating the local economy [8].

While there appears to be growing interest in medical malls, to-date there is no analysis on the current reach of these malls and the strengths and limitations of this model. To address these gaps, our study aimed to use quantitative and qualitative methods to: 1) describe the current reach of healthcare services in U.S. malls; 2) characterize the medical mall model and emerging trends; and 3) assess the potential of these facilities to increase healthcare access and serve the needs of low-income populations.

## Methods

To accomplish our study aims we used a mixed methods approach that included quantitative data analyses using a large database of U.S. malls, literature reviews, and qualitative interviews with key informants. This study was approved by RAND’s Institutional Review Board.

### Defining, identifying, and characterizing medical malls

#### Database searches: directory of major malls

To describe the current state of healthcare delivery at U.S. malls, we accessed the Directory of Major Malls (DMM), an online database representing the full universe of the approximately 7,200 large (200,000+ square feet) shopping malls currently in operation in the U.S. and Canada [9]. This database, primarily used by the retail industry, includes malls that are exclusively or primarily retail-focused and contains information on mall name and location, physical features, community demographics, and tenants. We limited our search to enclosed malls (n = 1,395) in the U.S. because healthcare tenants have historically occupied strip malls in large numbers and the interest in medical malls has focused on enclosed malls. Using keyword searches, we identified optometry (keywords: optic*, lens*, spectr*, and eye) and dental (keywords: dent*, orthod*, and dds) tenants. To identify other types of health care services, we searched tenants for the following keywords following consultation with database managers and review of the literature: clinic*, medic*, doctor, dr, health, dialysis, pediatr*, podiat*, chirop*, surg*, emergency, and urgent. We reviewed the names of the stores to eliminate misleading businesses such as “shoe doctor” that represented a retail tenant and medical equipment, non-surgical weight loss (e.g., Weight Watchers), and hearing aid stores. Our search uncovered select examples of alternative medicine providers (e.g., acupuncture, massage). We chose to exclude alternative providers because their business model is different and there are likely different issues with co-existing with retail stores. Also, practically, searching by keyword was an ineffective strategy for identifying the universe of providers. Many of these providers had non-descript names such as “Serenity” that could not be picked up through targeted keyword searches. For each mall, we classified healthcare tenants by type by reviewing information on each mall’s website and/or directly calling tenants to get verbal descriptions of services.

In addition to describing healthcare delivery at U.S malls more broadly, we also sought to use the DMM to identify medical malls and construct a database of these malls in the U.S. There is currently no formal definition of a medical mall. The term has been used to refer to large multi-specialty outpatient centers, multiple healthcare entities located in former enclosed or open-air (strip) malls, and mixed-use healthcare and retail facilities [10,11]. In searching the DMM, we defined a medical mall as an enclosed mall with at least five healthcare tenants. We chose this cut-off based on face validity and examining the distribution of the number of healthcare tenants in enclosed malls. A cut-off of five tenants appeared to be a natural threshold above which there were a relatively small number of malls.

#### Literature review

Using the following keywords: medic*, hospital, healthcare, delivery, shopping, mall, and retail, we searched databases of the peer-reviewed (PubMed, Google Scholar) and grey literature (New York Academy of Medicine Grey Literature Database, Google, Scopus, and OAIster). In addition, the database Lexis-Nexis was searched to identify news and magazine reports. Articles were considered relevant if they discussed a specific medical mall or discussed medical malls or mixed-use retail and healthcare facilities in general. If the article mentioned a medical mall not included in the DMM, we added it to our database of medical malls. 601 articles were included in the final review.

#### Typology of medical malls

The medical malls identified in the mall database and literature review were categorized into four possible categories: 1) fully healthcare-focused mall with no retail presence; 2) healthcare-focused mall with some retail tenants; 3) retail-focused with mall multiple medical tenants; and 4) mixed-use medical complex with retail components. Malls were considered category 1 if a healthcare entity bought or took over a former shopping mall and fully converted the space to healthcare services. Category 2 consisted of those malls that were healthcare-owned with some space leased to retail. In contrast, category 3 consisted of retail-focused malls that leased space to multiple medical tenants. Finally, category 4 represented a model of healthcare/retail blending where medical complexes or medical villages were originally built to include retail elements in the design. In addition to medical mall type, we collected data on the year each mall opened, its location, the median income in the surrounding community, and its tenant mix.

### Qualitative characterization of medical malls

#### Key informant interviews

We used the results of our literature review to identify potential stakeholders to recruit for key informant interviews. The specific research question that guided the qualitative component of the research was: what is the role as well as strengths and weaknesses of the medical mall model? Semi-structured interviews were determined to be the appropriate approach because they allowed us to discuss concepts in depth and to accommodate changes in the interview protocol depending on the emergence of new themes and specific areas of expertise of participants. We selected informants using purposive sampling to achieve maximum diversity; our aim was to capture a range of experiences and perspectives regarding the use of medical malls.

Potential informants were identified through literature reviews and snowball sampling. They were contacted by email and phone by the lead author. Sixty percent of contacted individuals agreed to participate. We do not know reasons for non-participation, because non-participants did not respond to interview requests (i.e., there were no direct refusal).

We conducted 45-minute phone interviews with representatives of three medical malls and five additional stakeholders representing healthcare organizations, healthcare consulting companies, and retail lease managers (Table 1). All participants were promised confidentiality and anonymity and provided oral consent. We used a semi-structured interview guide that varied slightly depending on the category of stakeholder, but in general included questions on strengths and limitations of the medical mall model (for patients, providers, retailers, and surrounding communities), the role of medical malls within the community, the potential of medical malls to reach low-income populations, and perspectives on future trends. We completed interview recruiting once saturation was reached (e.g., new interviews were not substantively adding to our analysis.) In general, we designed and executed the qualitative component of the study with consideration of RATS guidelines, which present a set of criteria to judge the quality of qualitative manuscripts [12].

**Table 1:** List of key informants

**Name**	**Location/Type of organization**
*Medical Mall Stakeholders*	
Biggs Park Mall	Lumberton, NC
Jackson Medical Mall	Jackson, MS
100 Oaks Mall/Vanderbilt University Medical Center	Nashville, TN
*Other Stakeholders*	
Simply Retail	Healthcare Consulting
Merchant Medicine	Healthcare Consulting
Sutter Health	Healthcare Organization
Shopping Center Group	Retail Lease Management

### Data analysis

We generated descriptive statistics on healthcare delivery at U.S. malls. In addition, qualitative data collected from the text of articles identified from the literature review and stakeholder interviews were thematically coded using MAXQDA 10 qualitative analysis software [13]. A hierarchically organized codebook was developed to identify and summarize themes and patterns [14]. Our codebook included a mix of *a priori* themes that followed the interview guide as well as themes that emerged during the interviews. We present the results from both the quantitative data analysis and thematic analysis below, including illustrative quotes to provide examples of key concepts. Quotes are used to represent the most frequently occurring concepts.

## Results

### Landscape of healthcare delivery at U.S. malls and medical malls

Of the 1,395 enclosed malls in the U.S., 870 (62.4%) leased space to one or more optometry tenants and 241 (17.3%) to one or more dental tenants. We identified 89 (6.4%) malls with other types of health care services. The most common types of non-dental or optometry tenants within the subset of malls with other types of health care services were primary care/general medical clinics (n = 35, 18.7%) and chiropractic offices (n = 29, 15.5%) (Table 2). Psychological services, acute care (e.g., urgent care and retail clinics), rehabilitation/physical therapy, and wellness services, each accounted for less than 7% of the non-dental or optometry healthcare tenants.

**Table 2:** Types of tenants in malls with healthcare tenants (n = 187)*

**Classification**	**Total healthcare tenants (n = 187)**	
	**N**	**%**
Primary care/General medical clinic	35	18.7
Chiropractic Office	29	15.5
Other specialty outpatient**	27	14.4
Psychology	13	7.0
Acute care	12	6.4
Rehabilitation/Physical therapy	10	5.3
Screening/wellness/health education	9	4.8
Elder services	7	3.7
Women’s health	6	3.2
Health department clinic/administration	6	3.2
Cosmetic dermatology/laser clinic	5	2.7
Dialysis	5	2.7
Surgery	5	2.7
Podiatry	4	2.1
Laboratory	4	2.1
Imaging	4	2.1
Substance abuse	2	1.1

*Summarizes results from malls (n = 89) that had healthcare tenants beyond dental and optometry.

**Includes cardiology, allergy, drug screening, pediatric speech and language therapy, ophthalmology, sleeping disorders, etc.

Through DMM searches and our literature review, we identified a total of 28 medical malls, with higher numbers found in the Southeast (10) and Midwest (7) as compared to the Northeast (5), West (4), and Southwest (2) (Table 3). We defined seven malls as category 1, six malls as category 2, ten malls as category 3, and five entities as category 4. In addition, we found 36 other facilities that characterized themselves as medical malls; however, it did not appear that any of these buildings were formerly retail spaces or currently shared space with retail.

**Table 3:** Existing or planned medical malls in the United States identified through literature review and directory of major mall searches

**Medical mall name or healthcare entity**	**Location**	**Retail name**	**Median household income**^**d**^	**Year opened**
**Category 1: Former shopping mall, now fully healthcare-focused and healthcare owned with no retail presence**				
Eastern Maine Healthcare	Bangor, ME	Westgate Mall	$37,510	1998
Appalachian Regional Healthcare	Hazard, KY	Wal-Mart	$26,534	2009
T.J. Health Pavilion	Glasgow, KY	Barren River Plaza	$34,145	In Development
HIMG Regional Medical Center	Huntington, WV	Wal-Mart	$49,233	2006
Kingwood Medical Center	Kingwood, TX	Deauxville Mall	$85,586	1991
St. Elizabeth Hospital	Beaumont, TX	Gaylynn Shopping Center	$29,651	1990s
Sutter Health	San Rafael, CA	Marin Square Shopping Center	$46,289	In Development
**Category 2: Former shopping mall, now healthcare-focused and healthcare owned with space leased to retail tenants**				
Dearborn Town Center	Dearborn, MI	Montgomery Ward Department Store	$34,122	2010
Holyoke Health Center	Holyoke, MA	Epstein’s Furniture, McAuslan & Wakelin	$11,310	2006
Station Medical Center	Altoona, PA	Station Mall	$26,076	2010
Elizabeth G. Means Medical Pavilion	Jacksonville, FL	Gateway Mall	$27,093	In Development
Boca Raton Regional Hospital	Boca Raton, FL	Oaks Plaza Shopping Center	$38,625	2000s
Jackson Medical Mall	Jackson, MS	Jackson Mall	$19,441	1997
**Category 3: Current shopping mall (retail/non-medically owned) with space leased to medical tenants**				
Various healthcare tenants	Evansville, IN	Washington Square Mall	$42,672	Unknown
Allied Physicians	Mishawaka, IN	University Commons/Kroger	$81,127	2011
Various healthcare tenants	Mankato, MN	Madison East Center	$43,983	Unknown
Various healthcare tenants	Frontenac, MO	Frontenac Grove	$155,042	Unknown
Various healthcare tenants	Batavia, NY	Genesee Country Mall	$33,958	Unknown
Various healthcare tenants	Tupelo, MS	Gloster Creek Village	$32,070	Unknown
Southeastern Regional Medical Center	Lumberton, NC	Biggs Park Mall	$26,442	2012
Vanderbilt University Medical Center	Nashville, TN	100 Oaks Mall	$31,942	2009
Various healthcare tenants	Oxnard, CA	Centerpoint Mall	$48,523	Unknown
Various healthcare tenants	Aiea, HI	Pearlridge Center	$61,765	Unknown
**Category 4: Intentionally designed mixed-use medical complex (healthcare owned) with retail components**				
North Memorial	Maple Grove, MN	The Grove	$89,015	2005
Metro Health Village	Grand Rapids, MI	Under Development	$60,612	In Development
Dartmouth-Hitchcock Medical Center	Hanover, NH	NA	$56,336	1991
McClellan Park Medical Mall	Anniston, AL	NA	$14,107	2008
Kaiser Permanente	San Leandro, CA	Albertson’s Distribution Center	$73,433	In Development

^d^Median household income for census tract in which mall is located. Data from 2010 American Community Survey 5-year estimates.

The 28 medical malls we identified offered a full range of outpatient services including primary care and specialty outpatient services (e.g., dialysis, psychological services, imaging, women’s health, acute care, and laboratory). Kingwood Medical Center, set in the former site of the Deauxville mall in Kingwood, TX, also offered inpatient rehabilitation services [15]. Overall, category 1 and 2 medical malls (repurposing of former malls) served communities with lower median household incomes compared to other categories of medical malls. The average median household income for the first two categories was approximately $35,000 compared to $56,000, and $59,000 for categories 3 and 4. For 21 of the malls, we were able to verify the date of opening. Among these, 14 (67%) opened since 2005 or were currently under development.

### Drivers of interest in medical malls

Just under half of the stakeholders we interviewed described the growth of medical malls as indicative of the growing interest of hospitals and health-systems in consumer-focused healthcare and an interest in “healthcare retail”. Healthcare retail refers to both bringing retail to a health care site (e.g. hospital campus) and offering healthcare services off-site in a retail space (e.g., in a mall or retail pharmacy). One stakeholder mentioned the Mayo Clinic’s presence in the Mall of the Americas as an example of healthcare’s growing interest in retail environments [16]. Although the Mall of the Americas is not an example of a medical mall, one stakeholder explained that in 2011, Mayo established a presence there to “learn more about the consumer and hone their expertise in a consumer-focused environment”. Stakeholders noted that the medical mall model appeals to mothers “who are both the major shoppers and healthcare decision-makers in their families”; therefore, there has been some emphasis on putting pediatric and women’s services in the mall.

### Model specifications

Stakeholders mentioned a number of key factors that were necessary for the success of model. According to two stakeholders, although healthcare tenants benefit from being co-located with retail stores, retail tenants are not essential. Stakeholders also noted that too much integration represents a problem for both retail and healthcare tenants. For healthcare tenants, too much integration can “tarnish their reputation and image”, especially in cases where the retail establishments cater to a different demographic (e.g., discount stores). The converse is also true. For example, restaurant customers may react negatively to the site of an acutely ill person entering an urgent care center. According to one stakeholder, “The GAP is never going to move into a mall that is dominated by healthcare players because of perceptions [that creepy crawly feeling you get when you are surrounded by sick people]”. Stakeholders agreed that mixed use spaces can thrive as long as separation is maintained. Two of the three medical malls we profiled discussed the need to maintain physical separation. In one medical mall, all healthcare tenants occupied the building’s second floor and in another, medical tenants were located in a strip center adjacent to the enclosed (exclusively retail) mall. One stakeholder also noted, however, that although retail is not critical to the success of a medical mall, retail elements could still be important for creating a comfortable environment and encouraging patients to be active participants in their healthcare.

### Strengths and limitations of the medical mall model

Stakeholders generally agreed that medical malls have numerous advantages for patients, providers, mall operators, and the wider community. For patients, medical malls increase access and convenience. Several interviewees used the term “one-stop-shop”, referring to a patient’s ability to run errands and attend to a variety of medical needs at the same time. In addition, one stakeholder discussed the benefits to family members who accompany patients undergoing time-intensive procedures such as chemotherapy. Friends and family appreciated having somewhere to walk as well as shop. Another stakeholder noted that a mall setting is familiar and thus, may be less intimidating than receiving care at a large academic medical center. There was some controversy over the true “convenience” of medical malls; stakeholders generally compared medical malls to a variety of sites for care and evaluated their convenience vis-à-vis different alternatives. For example, a medical mall with ample parking may be preferable to a large parking deck at a hospital, but it is inferior to driving up to a storefront to receive care at a clinic within a drug store in a strip mall. As three stakeholders pointed out, medical malls (that require you to walk long distances and navigate a complex mall directory) may be very convenient for a routine office visit but challenging for the provision of acute care. Furthermore, acutely ill patients are unlikely to take advantage of shopping opportunities.

One key advantage for providers is that placement in a medical mall can increase their visibility in the community since a clinic in a mall is likely to be seen by more potential patients than one in a medical office building. For mall operators, bringing in healthcare tenants is a great way to get “empty space leased” and increase traffic. In addition, healthcare tenants are attractive because they tend to have good credit and sign long-term leases. Finally, communities benefit from medical malls. One stakeholder noted that since the mall was renovated to include healthcare tenants, residents reported “feeling safer”. The introduction of a medical mall in an underperforming retail space can help revitalize the community. Finally, one stakeholder explained that communities often have emotional ties to the run-down mall in their town (that has had a historical presence) and they welcome opportunities to restore it.

While three stakeholders noted some limitations of the medical mall model, two stakeholders noted that there were not many disadvantages. One stakeholder cautioned against rushing to build medical malls because of lessons learned from retail clinics (i.e., many were not profitable): “You must be careful when going into a retail space. They are designed differently and customers behave differently in those environments. The healthcare sector has learned a lot from retail clinics regarding moving too quickly”. Furthermore, from the perspective of retailers, healthcare tenants take up valuable parking spaces for long periods of time and may in fact reduce demand for their services and merchandise. A key drawback that is not well understood is the costs associated with leasing space in a mall or converting an underperforming mall into a healthcare space. Two stakeholders and one media source explained that locating in a mall is in fact more expensive for providers than a traditional medical building but additional costs were offset by advantages such as increased traffic and visibility. One stakeholder decided not to move healthcare tenants into an existing mall because he was concerned about profitability.

### Role of medical malls in serving low-income communities

Two of the three malls we profiled served low-income populations, and one was explicitly created for the purpose of expanding access within a low-income community. If the focus of the mall is expanding access to reduce health disparities, stakeholders discussed the importance of obtaining buy-in from the community (e.g., creating a community advisory board), understanding its needs (e.g., ensuring the mall is on public transportation routes), and reflecting its demographics in the selection of providers and retail establishments. One mall used a foundation to promote its development, and it was generally felt that nonprofits and/or public-private partnerships are needed to extend reach to low-income populations. Stakeholders also generally agreed that partnerships were necessary because those in private business are “not going to open up a mall solely to improve access to care”. Several stakeholders explained that lessons learned from experiences with retail clinics applied to the use of malls for healthcare delivery. To support this point, they explained that market forces resulted in the placement of retail clinics in more affluent areas. According to one stakeholder, “If left to the market, medical malls- like retail clinics- are unlikely to increase access to low income individuals”.

Two of the medical malls in our study described the impact of taking over an underperforming (retail) mall on the economic wellbeing of the larger community. As explained by one stakeholder, “The medical mall transformed the community [brought in new businesses and reduced crime] although that is not what we originally intended to do”. Another stakeholder observed that the introduction of a medical mall could be part of a comprehensive urban renewal strategy.

### Future trends for medical malls

Of the five stakeholders who commented on the future of the medical mall model, three enthusiastically claimed that medical malls were poised for growth and felt existing success stories had generated interest from domestic and international players. Others expressed skepticism and noted that too much is unknown related to the impacts of the Affordable Care Act, patient preferences, and the degree to which technology will support e-visits and telemedicine to predict the future of the medical mall. In general, numerous stakeholders noted that the success of this particular model was community-dependent. As one stakeholder noted, “A medical mall may work somewhere but flop somewhere else”. Factors such as the design of the mall, demographics of the community, and alternatives for care all affect the likelihood of success.

## Discussion

Medical malls have received significant media attention, and experts have described the potential of healthcare to save the dying American mall [17]. The stakeholders we interviewed agreed that medical malls are poised for growth, and the majority of malls we identified opened in the last five years (or were scheduled to open in the near-term), suggesting that they represent a new trend in healthcare delivery; however, our review found that the current role of healthcare in malls in the U.S. is limited. At present, 6% of large, enclosed shopping malls include healthcare tenants (other than optometry and dental) such as primary care practices. Using our more narrow definition, there are 28 medical malls across the U.S., which is approximately half the number estimated by grey literature sources [6,18].

The stakeholders we interviewed highlighted numerous benefits of medical malls as compared to more traditional sites for healthcare delivery such as medical office buildings. These included the ability to provide “one-stop” shopping and convenience for patients and greater visibility for providers. Medical malls, furthermore, reflect the growing interest among healthcare providers in creating a more consumer-centric experience. However, we found that only some types of healthcare services may be suited for a medical mall. Primary care and specialty services which require routine visits are ideal. Women’s health and pediatrics are also attractive given that women are more likely to take advantage of the shopping opportunities at the mall. In contrast, acute care services may not be well suited to a mall setting. A note of caution is also warranted. While there is great interest in medical malls, there is also the possibility that enclosed malls will fail to attract healthcare providers for the same reasons that they increasingly fail to appeal to retailers.

Proponents argue that medical malls like the Jackson Medical Mall improve access to low-income communities and can serve as an engine of local economic growth. Unfortunately, it appears that malls like Jackson remain the exception, not the rule. Most medical malls in the U.S. serve middle to high-income populations. The stakeholders we interviewed believed medical malls would not open on their own in low-income communities. Rather, it is critical to engage the community, creating partnerships and understanding community concerns to promote trust, reduce crime, and enhance economic development.

There are several limitations to our study. First, our DMM search strategy could fail to identify some medical specialties not represented among our keywords as well as healthcare tenants with general, non-descriptive names. Second, because there is no standard definition of medical mall, we had to develop and apply a novel definition for the purposes of identifying and characterizing medical malls. Similarly, very little is written about medical malls in the peer- reviewed literature; thus, we must draw conclusions about medical malls from a comprehensive search of grey literature and from stakeholders who shared anecdotal evidence. We confronted this challenge by employing a mixed methods design and through triangulation of research methods and sources. Finally, we did not interview patients for this study and thus cannot directly represent their perspectives; however, three stakeholders referenced the results of focus groups and surveys (conducted as market research) with patients on their experience with and impressions of the local medical mall.

## Conclusion

While our exploratory study uncovered many new insights about healthcare delivery in medical malls, there is a great deal that we do not understand about this topic. For example, the success of a particular medical mall seems to be dependent on a range of (thus far unspecified) community factors such as alternatives for care, the mix of tenants, and the populations served. While stakeholders argue that the mall is not suited for the provision of acute care and makes more sense for primary and specialty care that is received on a routine basis, there is no empirical evidence to support this claim other than the fact that retail clinic operators have found enclosed mall locations to be less profitable than alternative locations over the past several years. It should be noted that although this review was limited to medical malls in the U.S., there are numerous examples of medical malls across the globe, most notably in Asia [19,20]. The Jackson Medical Mall in particular has served as a model for developers abroad [8]. Future research should explore whether international experiments with this model can provide lessons learned for the U.S.

Future research is also needed to understand the precise conditions that contribute to the success and impacts of medical malls on costs and quality of care. At present, medical malls only have a great deal of *potential* to improve access to care, transform the patient experience, reach low income individuals, and help “save” the American mall.

## Competing interests

The authors declare that they have no competing interests.

## Authors’ contributions

LUP and RC conducted the literature review and interviews, analyzed interview and secondary data, and drafted the manuscript. AM helped to design the study, secured funding for the work, and revised the manuscript. All authors read and approved the final manuscript.

## Pre-publication history

The pre-publication history for this paper can be accessed here:

http://www.biomedcentral.com/1472-6963/13/471/prepub
